# Evidence of Multiple Inseminations in the Field in *Aedes albopictus*

**DOI:** 10.1371/journal.pone.0042040

**Published:** 2012-08-15

**Authors:** Sebastien Boyer, Celine Toty, Maxime Jacquet, Guy Lempérière, Didier Fontenille

**Affiliations:** 1 MIVEGEC, Institut de Recherche pour le Développement (IRD) UMR 224, Centre National de la Recherche Scientifique (CNRS 5290), Universités Montpellier 1 and 2, Montpellier, France; 2 Centre de Recherche et de Veille sur les Maladies Emergentes dans l'Océan Indien (CRVOI), Sainte Clotilde, Réunion; Kansas State University, United States of America

## Abstract

Studies on the biology and mating behaviour of male mosquitoes are of major importance in a frame of a Sterile Insect Technique which could be used against mosquito vector species. Most particularly, the assumption of possible multiple inseminations in mosquito species must be investigated in order to optimize alternative mosquito control methods (Sterile Insect Techniques with genetically modified mosquitoes, cytoplasmic incompatibility, radiation…). The occurrence of multiple insemination events was investigated after 2 field samplings of *Aedes albopictus* (Diptera: Culicidae) in La Reunion Island using microsatellite markers. Respectively, 14 and 13 females after the first and the second sampling laid eggs. Seven wild females out of the 27 laying females were found with a progeny involving more than one father. This result is important for the new alternative mosquito control methods and raises the importance of pre- and post-copulatory competition.

## Introduction

*Aedes albopictus* Skuse (Diptera: Culicidae) is a mosquito species of major sanitary importance. It is involved in the transmission of several diseases and especially studied for its role in arbovirus transmissions such as Dengue and Chikungunya [1]–[4]. Its current involvement in Chikungunya diseases from Africa to Asia [5]–[7], its high vector competence combined with an efficient spreading behaviour, confirm the danger represented by this species. Furthermore, beyond the real risk of disease transmission, in many areas *Ae. albopictus* imposes a severe biting nuisance affecting the quality of life of inhabitants in infested areas.

Current control methods against this vector are not efficient enough. Sterile insect techniques (SIT) are under development and could lead to the decrease of mosquito densities. SIT is considered as a species-specific and environmentally-safe method. Its strategy is based on repeated releases of sterilized males [8]–[9]. Genetically Modified Mosquitoes (GMM) [10], cytoplasmic incompatibility (CI) [11] and classical SIT using radiation are currently the most developed and sometimes used techniques in SIT. GMM refers to a modification of the genome of individuals from the species. This technique has been developed on A*edes aegypti* species [12] with releases in the Caymans Islands in 2010 [10]. CI refers to the ability of *Wolbachia* to the inviability of embryos produced by fertilisation by sperm from *Wolbachia*-infected males of eggs from uninfected females, which allows *Wolbachia* to invade and spread through insect populations. Infection with certain strains of *Wolbachia* can reduce the ability of the host insect to transmit dengue virus. A recent small field trial in Australia showed that one such strain can invade a wild *Aedes aegypti* population [13].. SIT refers to the classical radiation of male pupae or adults before their release in the field. It has already been used as a control method in Italy since 2007 [14].

SIT can be considered as a viable approach in vector control, and mosquito biology, ecology, behaviours, dispersal, genetics, competitiveness, fitness of sterilised males have to be investigated consequently. In a frame of a future SIT strategy in La Reunion Island, the biology of immature stages [15], blood feeding behaviour of females [16], flight behaviour, dispersal of males [17], estimates of population size, mating behaviour of males [18] and its origin [19] have already been studied. Nowadays, in the context of SIT programmes, it is important to know whether the progeny from wild-caught females come from a single male, or whether more than one male have contributed in sperm donation.

## Materials and Methods

### Mosquito population

*Aedes albopictus* mosquitoes were collected in the field when landing on human volunteers during 2 field catches on the 10^th^ February and on the 18^th^ April 2011, in the urban area of Saint-Denis (20°55′13.80″S; 55°30′52.87″E). No specific permissions were required for this location, which is not a private property. Female mosquitoes were collected before biting in the field by two volunteers using man-made mouth aspirators. No specific ethical clearance permit is required for the mosquito collection by volunteer landing catches in La Reunion. As soon as female *Ae. albopictus* land on human body, they were aspirated, collected and transferred to the laboratory. 20 females and 20 females were caught during the first and the second field samplings. No specific permits were required for the described field studies. This field study did not involve endangered species.

### Egg laying of females

Females from the field were individually put into plastic cups and fed on cotton with a 10% sugar solution. After a blood meal, a seed paper was added in each cup in order to collect eggs. Females captured during the first field sample were allowed to lay eggs one time. A second blood meal was given to the females from the second field sample, in order to obtain a second batch of eggs. 14 females of the first field sample and 13 females from the second sample laid eggs. *Aedes* females were then kept at −20°C. Eggs from the same batch and from the same female were put into water for hatching. Third instar larvae were individually collected and kept at −20°C.

### DNA extraction, amplification

Genomic DNA of all samples (larvae and adults) was extracted following a modified version of the protocol proposed by Edwards [20]. DNA extraction was performed individually on 955 larvae and 27 females using 200 µL of CTAB 2% (Cetyl trimethyl ammonium bromide) for the crushing step, followed by a 5 min bath at 65°C. Chloroform was then added volume to volume to separate the components into both hydrophilic and hydrophobic phase. After 5 min centrifugation at 12000 rpm, the hydrophilic phase was kept and DNA precipitated with 2-propanol volume to volume. After 15 min centrifugation at 12000 rpm, the surpernatant was discarded and the pellet was washed with 70% ethanol before a last 5 min centrifugation at 12000 rpm. Ethanol was then discarded and the pellet dried in the speed vac. The pellet was finally resuspended in water during midday at room temperature and stored at −20°C for molecular analysis.

### Genotyping

Genetic variability was assessed using two microsatellites markers. The marker A9 was developed with *Ae. albopictus* and is highly polymorphous [21] and AEDC was developed with *Aedes aegypti* and fit very well with *Ae. albopictus* [22] (Table 1). Forward primers were fluorescently end-labelled (PET or 6-FAM; Applied Biosystems, Foster City). DNA amplification was conducted on a 96-well GeneAmp PCR System 9700 (Applied Biosystems) using the Qiagen multiplex PCR kit. PCR was performed in a 10 µl volume containing 5 µl Master Mix 2X, 1 µl DNA and each primers pairs at 0.30 µM. The PCR reactions were initially denatured at 95°C for 15 min to activate the Taq polymerase, followed by 35 cycles of amplification: 94°C for 30 seconds, 60°C to 50°C for the 10 first cycles (decrease 1°C per cycle) for 1 min 30 sec and 72°C for 1 min 30 secs and 25 cycles of amplification at 94°C for 45 sec, 56°C for 1 min 30 sec and 72°C for 1 min and 30 sec and finally extended at 60°C for 30 min. PCR were confirmed after electrophoresis conducted on individuals randomly chosen in each plate (PCR gels are illustrated in Figure S1 and Figure S2). 8 µl of these individuals in addition to 2 µl of loading buffer was loaded in a 4% agarose gel stained with ethidium bromide. The gel was run at 140 V at 220 mA during 1.5 h. 1 µl PCR product was mixed with 10.85 µl of Hi-Di formamide (Applied Biosystems) followed by the addition of 0.15 µl of GeneScan 500 LIZ Size Standard (Applied Biosystems) for the reproducible sizing of the fragments, and then analyzed using 3130 Genetic Analyzer (ABI Prism 700) (Applied Biosystems). Allele sizes and genotypes were defined using GeneMapper software (Electrophoregram gels are illustrated in Figures S3 and Figure S4).

**Table 1:** Characteristics of microsatellites used for the assessment of the genetic structure of *Aedes albopictus* in La Réunion Island.

Marker	GenBank number	SSR motif	Primer sequences (5′-3′)	Size (bp)	N_A_	F*_is_*	P
A9	DQ366022	(AC)_4_GCAT(AC)_2_TC(AC)_8_CCAA(AC)_2_CG(AC)GT(AC)C(AC)AT(AC)	F: 5′- TGGGACAAGAGCTGAAGGAT-3′	142–162	11	0.2187	0.0766
			R: 5′-CTCGTTCTCTACTCTCTCCGTT-3′				
AEDC	CT58313	(GTA)6(ACG)(GTA)3	F: 5′-TGCAGGCCCAGATGCACAGCC-3′	220–226	5	0.0954	0.8245
			R: 5′-TCCGCTGCCGTTGGCGTGAAC-3′				

N_A_, number of alleles.

F*_is_*, imbreeding coefficient (Weir & Cockerman).

P, probability for departure from Hardy-Weinberg proportions.

### Data analysis

Data were analyzed using GeneMapper software for fragment analysis (Applied Biosystems) to derive microsatellite allele sizes and genotypes. The number of potential father(s) was determined and data were analysed using the software GERUD for the reconstruction of parental genotypes from half-sib progeny arrays with known or unknown parents [23]. GERUD 2.0 software allows to determine the minimum number of males contributing to each mother-offspring array. Because GERUD uses exclusion to estimate the number of male genotypes contributing to a given progeny array, estimates using this program are considered conservative and should never overestimate the number of “father” [23]. Genetic variability parameters, estimation of the inbreeding coefficient and Hardy-Weinberg tests were conducted using GENEPOP software version 4.0.11 [24].

A Chi-square test was carried out in order to compare the differences in the numbers of eggs coming from the different males between the 1^st^ and the 2^nd^ egg laying of the same female (JMP 8.0 software, Statsoft inc., Paris, France).

## Results

**Figure 1:**
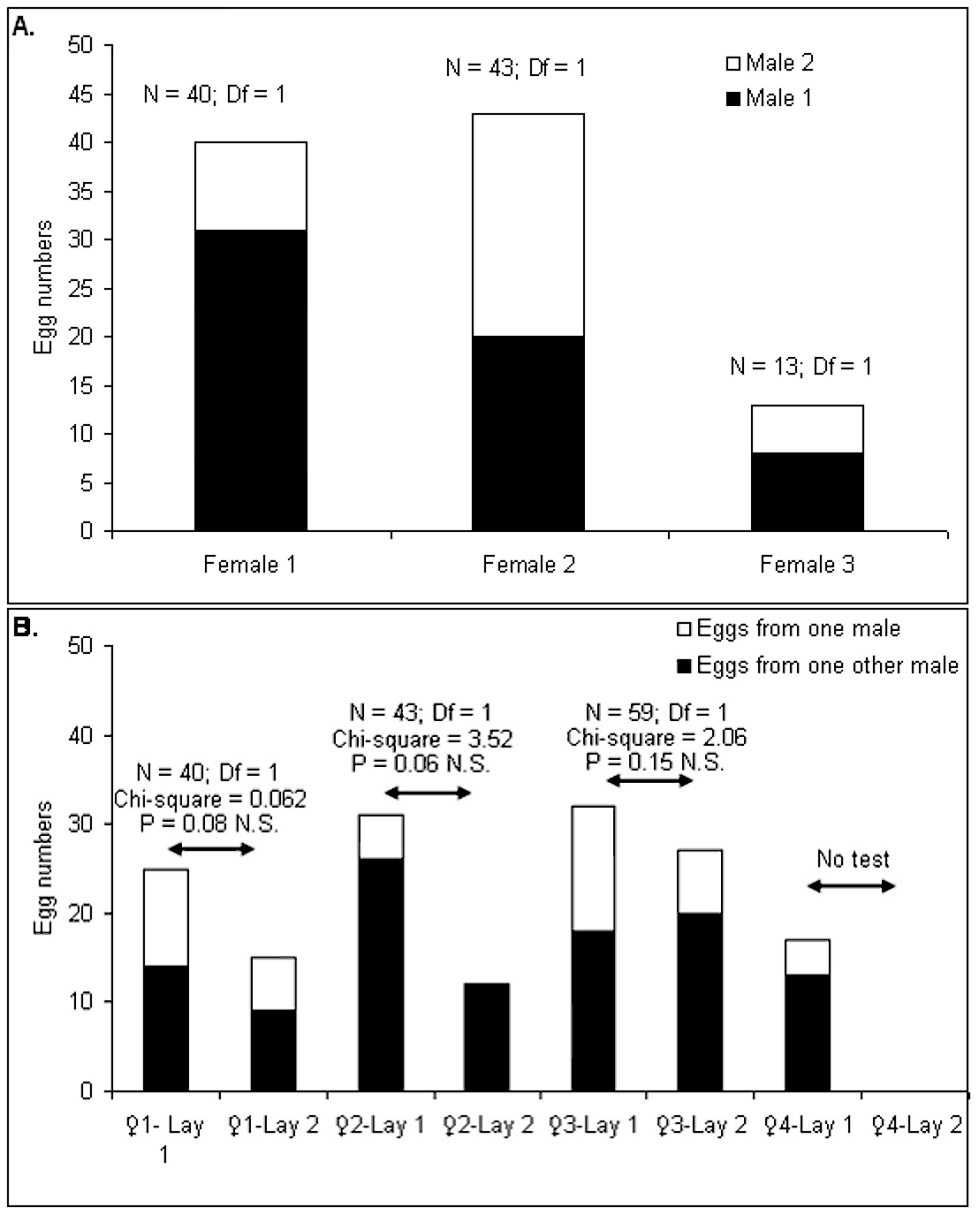
1A. Number of progeny (L3 larvae) from the 3 females inseminated by at least two males during the 1^st^ field sampling. Differences in color (white/black) represent the distribution in the number of larvae coming from one male or from more than one male. 1B. Number of progeny (L3 larvae) from the 4 females inseminated by at least two males during the 2^nd^ field sampling. Differences in color (white/black) represent the distribution in the number of larvae coming from one male or from more than one male. A Chi-square test was performed to test the distribution of sperm coming from male 1 or male 2 between the first and the second egg laying. The 4th female laid only one time. N = number of larvae;, Df = Degree of freedom; N.S. = Non Significant.

Genetic analysis of the 955 larvae coming from the females *Aedes albopictus* collected in the field showed that multiple inseminations occurred in natura (Table 2). Within the 1^st^ field catch, at least 3 pools of progeny out of the 14 tested were issued from a multiple insemination event (Table 2, Figure 1A, Table S1). These results of multiple inseminations were confirmed with a second field catching occurring two months later: 13 females from the field laid eggs and at least 4 pools of progeny were also issued from a multiple insemination event. Four females out of the 13 females issued from the 2^nd^ field catching were able to have a 2^nd^ egg laying (Figure 1B). Within the 2^nd^ egg laying, 3 pools of progeny from the same females with multiple inseminations during the first egg laying, showed multiple insemination profiles similar to those of the 1^st^ egg laying (Figure 1B); the 4^th^ female hatching eggs only one time.

**Table 2:** Number of multiple inseminations in 27 female *Aedes albopictus* from La Reunion Island.

	1^st^ sample	2^nd^ sample
Number of female analyzed	14	13
Number of genotyped offspring's larvae	414	541
Single father^1^	11	9
At least two fathers^2^	3	4

1 Number of females with no more alleles detected in pools of progeny than would be consistent with the hypothesis that all shared a single father.

2 Number of females with alleles detected in pools of progeny consistent with the hypothesis of at least two fathers.

## Discussion

Results of our field investigations did not fit with the generally accepted hypothesis that female mosquitoes are inseminated in the field only by one male. Despite results of potential multiple matings and multiple inseminations under laboratory conditions, most authors have rejected the hypothesis of polyandry in the field, arguing that suitable partners were at much lower densities in the field, and the cost and risk of a mating event was more significant. [25]–[26]. They were observed with encaged *Culex pipiens* within 48 h after the first mating under laboratory conditions [25]. Multiple inseminations were also observed with encaged *Culex tarsalis* [26]. Polyandry was observed in *Anopheles* species under laboratory conditions: occurrence of multiple inseminations was demonstrated in *Anopheles albimanus* under laboratory conditions where 0.6% female ovipositing the 2^nd^ time used the second male sperm [27]. Multiple inseminations were observed in *Aedes aegypti* in semi-field conditions within 48 hours, with an observed occurence frequency of 7% [28]. Multiple matings but no multiple inseminations were observed in the field in *An. freeborni* [29]. Authors hypothesized that females mated more than once when their first mate failed to impose monogamy [29]. There was no observation of multiple fertilizations in *An. quadrimaculatus* under laboratory conditions [30]. In *An. gambiae*, multiple inseminations were estimated around 2.5% under field conditions [31].

In *Ae. albopictus* we observed that multiple inseminations occur in the field species and also that the fertilization of eggs could be done by the sperm issued from several males. We were able to confirm the evidence of multiple inseminations using microsatellites. Considering the low number of microsatellite markers used in our study, we could suspect that the observed 26% of multiple insemination events as well as the number of different inseminations per female were underestimated, despite the occurrence of 3 alleles for marker 1 and 11 alleles for marker 2 (Table 1). The males and females belonging to the same population might carry the same alleles than the females they mated with, consequently hiding differences that could not be detected within our genotyping experiment. Despite this likely underestimation, the high number of multiple insemination events observed in the field raises the question of the frequency of this behaviour in natura.

There was no difference between the progeny of the first two batches of eggs. This result highlights the dearth of information concerning the use of different sperms by female mosquito. It also raises the question of sperm competition between wild males and between sterile and wild males. With the highlighting of multiple mating events occurring in the field, the sperm competition between sterile and wild mosquitoes needs further investigations. Recent works on the sperm in *Ae. aegypti* in relationship with the male size [32] or on the effect of radiation on *An. arabiensis* sperm [33] have been carried out but the sperm competition and the post-copulatory effects in *Aedes* species have not been studied yet. The impact of polyandry on SIT-type methods brings into play post-copulatory effects that would not otherwise be relevant. If females only mate once, then one would wish to be certain that female refractoriness to remating is fully or equally induced by the sterile male; if so then post-copulatory effects are irrelevant. If females could mate more than once then sperm precedence, competition, refractoriness to remating (if any) become potentially relevant - in other words, the whole gamut of post-copulatory effects. As long as the sperm of released sterile male *Ae. albopictus* is able to compete with the sperm of wild males in fertilizing eggs, polyandry should not be a problem in SIT control. The net effect on the targeted population will then depend on the proportion of matings by released sterile males. The evidence of multiple inseminations being part of the field behaviour in *Aedes albopictus* is major issue and of high relevance to improve the efficiency of SIT male programs.

## Supporting Information

Figure S1 Example of result obtained after migration of one PCR realized with A9 marker on the progeny from two different *Aedes albopictus* females.
(JPG)

Figure S2 Example of result obtained after migration of one PCR realized with AEDC marker on two different *Aedes albopictus* females.
(JPG)

Figure S3 Example of an electrophoregram obtained after PCR realized with A9 marker on an *Aedes albopictus* individual. The screenshot from GeneMapper software represents two electrophoregrams of an heterozygous individual (marker A9; the below image being a zoom of the image above).
(JPG)

Figure S4 Example of an electrophoregram obtained after PCR realized with AEDC marker on an *Aedes albopictus* individual. The screenshot from GeneMapper software represents two electrophoregrams of an homozygous individual (marker AEDC; the below image being a zoom of the image above).
(JPG)

Table S1 Genotyping of *Aedes albopictus* individuals studied with microsatellite markers A9 and AEDC. The female and offsprings genotypes were observed with the electrophoregram. The male genotype was obtained with Gerud 2.0.
(DOC)
