# Supplementary figures and images for: Evidence of Multiple Inseminations in the Field in Aedes albopictus
Source: PLoS One. 2012 Aug 15;7(8):e42040. doi: 10.1371/journal.pone.0042040 (PMC3419715; doi:10.1371/journal.pone.0042040)

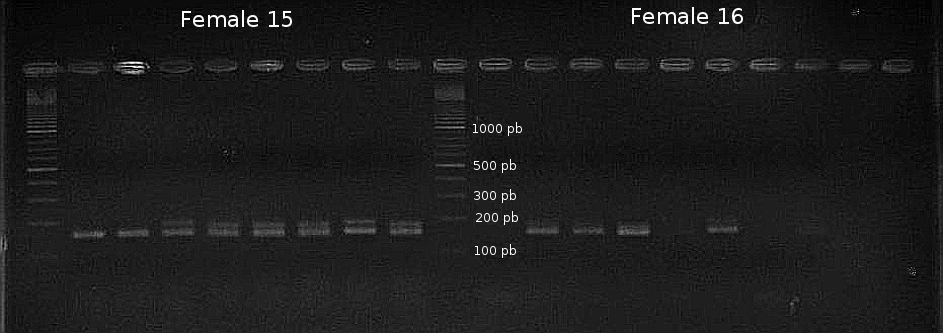

Supplement: Figure S1 — Example of result obtained after migration of one PCR realized with A9 marker on the progeny from two different Aedes albopictus females. (JPG) [file pone.0042040.s001.jpg]

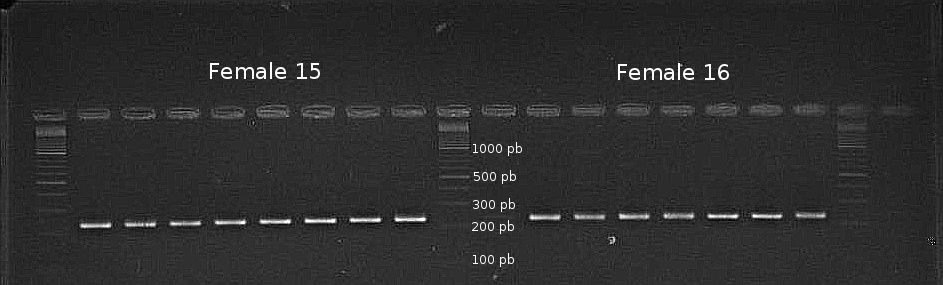

Supplement: Figure S2 — Example of result obtained after migration of one PCR realized with AEDC marker on two different Aedes albopictus females. (JPG) [file pone.0042040.s002.jpg]

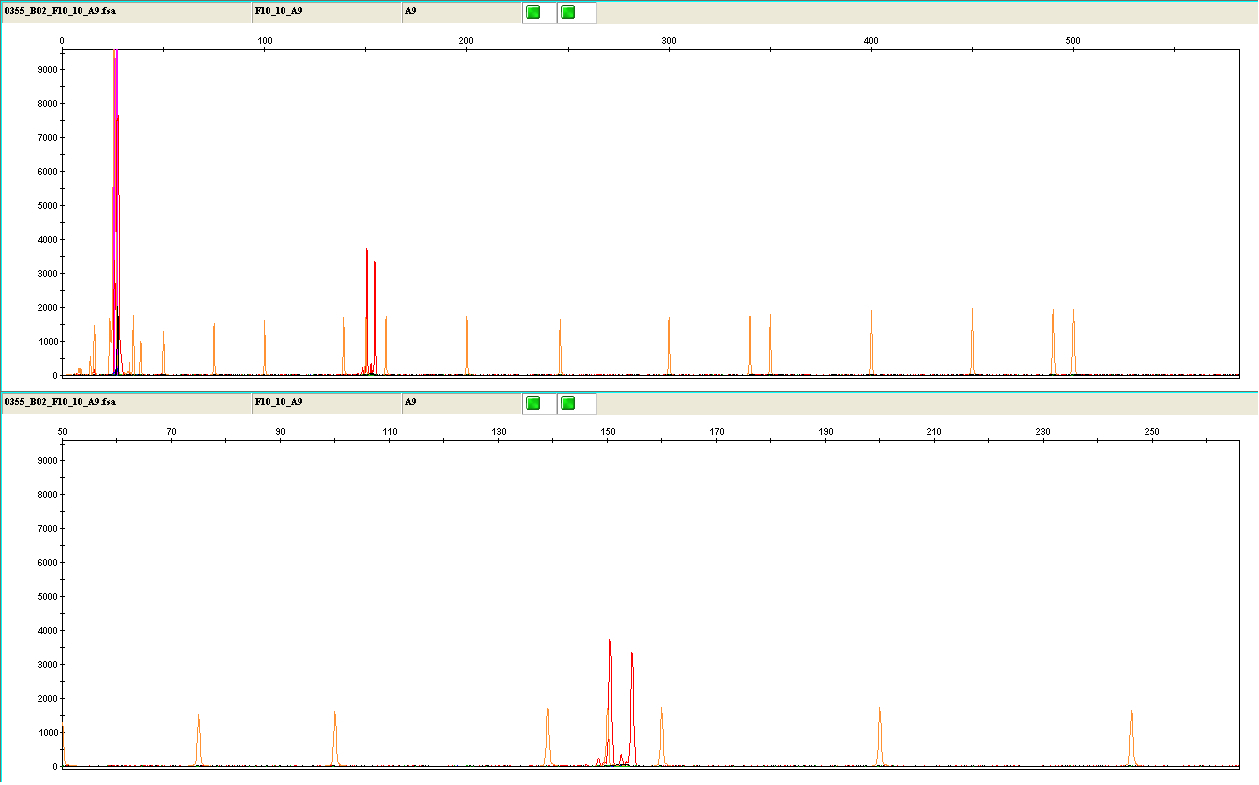

Supplement: Figure S3 — Example of an electrophoregram obtained after PCR realized with A9 marker on an Aedes albopictus individual. The screenshot from GeneMapper software represents two electrophoregrams of an heterozygous individual (marker A9; the below image being a zoom of the image above). (JPG) [file pone.0042040.s003.jpg]

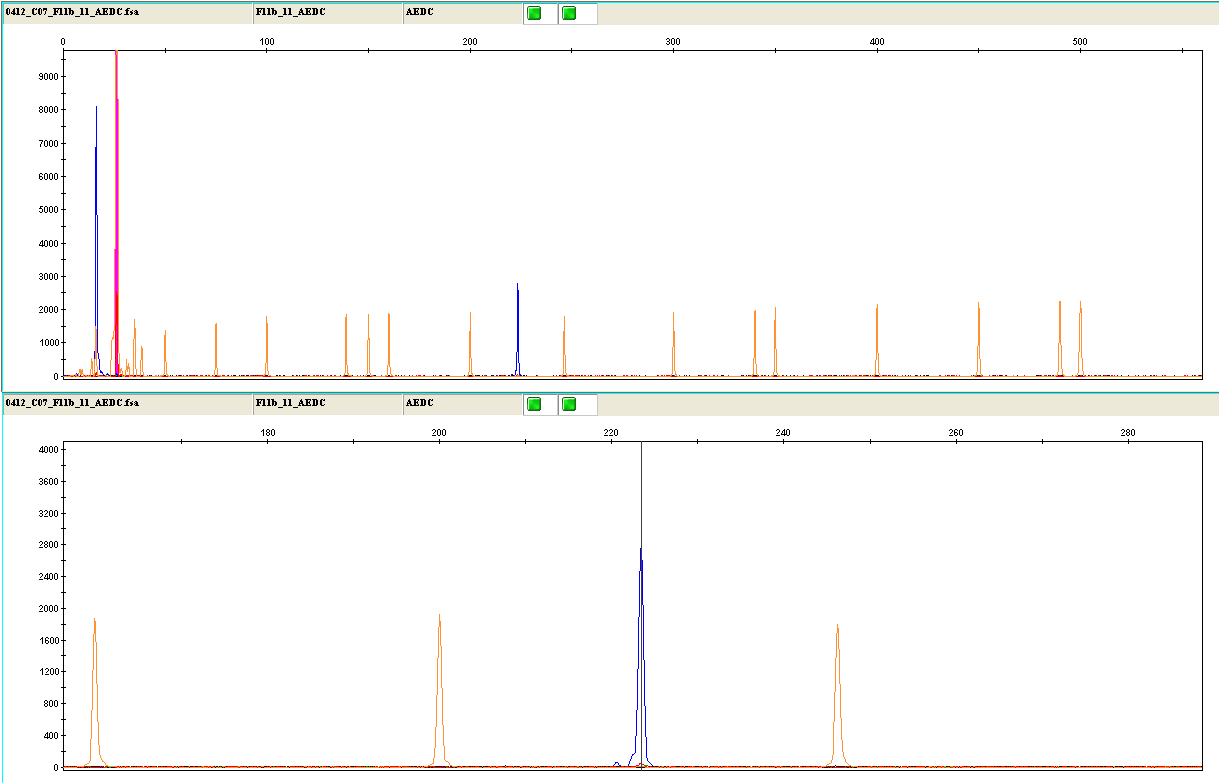

Supplement: Figure S4 — Example of an electrophoregram obtained after PCR realized with AEDC marker on an Aedes albopictus individual. The screenshot from GeneMapper software represents two electrophoregrams of an homozygous individual (marker AEDC; the below image being a zoom of the image above). (JPG) [file pone.0042040.s004.jpg]
