# Supplementary material for: Evidence of Multiple Inseminations in the Field in Aedes albopictus
Source: PLoS One. 2012 Aug 15;7(8):e42040. doi: 10.1371/journal.pone.0042040 (PMC3419715; doi:10.1371/journal.pone.0042040)
Supplement: Supplementary file 2 [file pone.0042040.s005.doc]

Family (n progeny)	locus	female	offspring observed genotypes					Male genotype	
F12 (19)	A9	142/148	142/146 (10)	146/148 (5)	142/142 (4)			146/142	
	AEDC	220/223	220/223(8)	223/223 (11)				223/223	
F24 (38)	A9	152/152	148/152 (19)	152/152 (19)				148/152	
	AEDC	223/226	220/226 (17)	226/226 (4)	223/226 (10)	220/223 (7)		220/226	
F25 (43)	A9	150/154	150/150 (15)	144/154 (8)	154/154 (5)	144/150 (15)	150/150 (15)	150/144	154/154
	AEDC	220/223	220/220 (27)	220/223 (16)				220/220	220/220
F26 (17)	A9	150/152	150/150 (5)	152/152 (4)	146/150 (5)	146/152 (3)		150/146	152/152
	AEDC	220/223	220/223 (11)	223/223 (6)				223/223	223/223
F27 (59)	A9	154/160	150/154 (10)	150/160 (28)	154/154 (14)	160/160 (7)		150/150	154/160
	AEDC	220/223	220/226 (16)	223/223 (10)	220/223 (19)	223/226 (14)		226/223	226/223
F17 (40)	A9	142/150	142/142 (13)	150/154 (6)	142/154 (11)	150/150 (10)		142/150	154/154
	AEDC	223/223	223/223 (40)					223/223	223/223
F18 (41)	A9	154/154	152/154 (23)	154/158 (18)					
	AEDC	220/223	220/223 (26)	220/220 (15)					
F19 (54)	A9	152/152	150/152 (31)	152/156 (23)				150/156	
	AEDC	223/223	223/223 (54)					223/223	
F20 (33)	A9	148/154	148/154 (8)	154/162 (9)	148/162 (5)	154/154 (11)		154/162	
	AEDC	220/223	220/223 (21)	223/223 (12)				223/223	
F21(24)	A9	unknow	142/148(9)	146/148 (7)	142/146 (5)	148/158 (3)		*Mother*	*Father1*
	AEDC	unknow	223/223 (14)	220/223 (9)	220/220 (1)			*148/142*	*146/158*
								220/223	*220/223*
F22 (30)	A9	154/160	158/160 (11)	154/158 (8)	144/154 (7)	144/160 (4)		158/144	
	AEDC	223/223	223/223 (30)					223/223	
F23 (46)	A9	152/158	152/156 (17)	156/158 (8)	146/152 (11)	146/158 (10)		156/146	
	AEDC	223/223	223/223 (46)					223/223	
